# Screening of ulcerative colitis biomarkers and potential pathways based on weighted gene co-expression network, machine learning and ceRNA hypothesis

**DOI:** 10.1186/s41065-022-00259-4

**Published:** 2022-11-23

**Authors:** Ying Li, Mengyao Tang, Feng Jun Zhang, Yihan Huang, Jing Zhang, Junqi Li, Yunpeng Wang, Jinguang Yang, Shu Zhu

**Affiliations:** 1 Shandong University of Traditional Chinese Medicine, College of Traditional Chinese Medicine, Jinan, China; 2 Shandong University of Traditional Chinese Medicine, The First College for Clinical Medicine, Jinan, China; 3 Shandong University of Traditional Chinese Medicine, College of Innovation and Research of Traditional Chinese Medicine, Jinan, 250000 China; 4 Affiliated Hospital of Shandong University of Traditional Chinese Medicine, Department of Gastroenterology, Jinan, China

**Keywords:** Ulcerative colitis, Machine learning, Bioinformatics analysis, WGCNA, Biomarkers, ceRNA

## Abstract

**Background:**

Ulcerative colitis (UC) refers to an intractable intestinal inflammatory disease. Its increasing incidence rate imposes a huge burden on patients and society. The UC etiology has not been determined, so screening potential biomarkers is critical to preventing disease progression and selecting optimal therapeutic strategies more effectively.

**Methods:**

The microarray datasets of intestinal mucosal biopsy of UC patients were selected from the GEO database, and integrated with R language to screen differentially expressed genes and draw proteins interaction network diagrams. GO, KEGG, DO and GSEA enrichment analyses were performed to explore their biological functions. Through machine learning and WGCNA analysis, targets that can be used as UC potential biomarkers are screened out. ROC curves were drawn to verify the reliability of the results and predicted the mechanism of marker genes from the aspects of immune cell infiltration, co-expression analysis, and competitive endogenous network (ceRNA).

**Results:**

Two datasets GSE75214 and GSE87466 were integrated for screening, and a total of 107 differentially expressed genes were obtained. They were mainly related to biological functions such as humoral immune response and inflammatory response. Further screened out five marker genes, and found that they were associated with M0 macrophages, quiescent mast cells, M2 macrophages, and activated NK cells in terms of immune cell infiltration. The co-expression network found significant co-expression relationships between 54 miRNAs and 5 marker genes. According to the ceRNA hypothesis, NEAT1-miR-342-3p/miR-650-*SLC6A14*, NEAT1-miR-650-*IRAK3,* and XIST-miR-342-3p-*IRAK3* axes were found as potential regulatory pathways in UC.

**Conclusion:**

This study screened out five biomarkers that can be used for the diagnosis and treatment of UC, namely *SLC6A14, TIMP1, IRAK3, HMGCS2,* and *APOBEC3B*. Confirmed that they play a role in the occurrence and development of UC at the level of immune infiltration, and proposed a potential RNA regulatory pathway that controls the progression of UC.

**Supplementary Information:**

The online version contains supplementary material available at 10.1186/s41065-022-00259-4.

## Introduction

Ulcerative colitis (UC) and Crohn’s disease belong to inflammatory bowel diseases (IBD), as a refractory intestinal inflammatory disease. UC has the characteristics of continuous, inversion, and non-specificity. The involved sites are mainly the colonic mucosa and submucosa, and the disease site can extend from the rectum to the proximal colon [1]. According to statistics, the incidence of UC in Asia has gradually increased in recent years, from 7.6/100,000 to 14.3/100,000, and the prevalence rate has also increased from 2.3/100,000 to 63.6/100,000 [2, 3]. Based on its large population, China has become one of the regions with the fastest growth rate of UC incidence and the heaviest burden of UC, with the number of cases rising to 3.08 times that of 1981–1990 in 1991–2000 alone [4]. The etiology of UC is unclear, but it is closely related to autoimmune dysfunction. The onset of UC is very insidious, and the early clinical manifestations are mainly abdominal pain, diarrhea, and even pus and blood in the stool, which is easily confused with other diseases such as infectious colitis and hemorrhoids. Clinical treatment is mainly based on long-term and dynamic monitoring of objective inflammation, and corresponding symptomatic treatment is made according to the patient’s condition. Although intestinal endoscopy is a key method for diagnosing and monitoring the disease, due to the invasiveness and inconvenience of this method, it is not easy to accept for some UC patients and potential disease populations. Therefore, it is necessary to search for new UC marker genes to optimize the diagnosis, monitoring and treatment of UC. In addition, new marker genes can deepen the understanding of UC disease and provide new directions for elucidating disease mechanism research and developing new drugs.

Predictive, preventive, and personalized medicine programs will be the general trend of medical development in the future. As a new means, machine learning can help us integrate information from multiple datasets and screen out biomarkers with clinical diagnostic and therapeutic value to help clarify the pathogenesis of diseases. Competitive endogenous network (ceRNA) is a mechanism hypothesis that has attracted much attention in recent years, which reveals the competitive relationship between a kind of RNA, such as messenger RNA (mRNA), long non-coding RNA (lncRNA), and microRNA (miRNA). Research on ceRNA networks in the field of IBD is emerging. Liu, Li et al., by studying lncRNA expression in mouse intestinal epithelial cells, proposed that lncRNA NONMMUT143162.1 and LncRNA ENSMUST00000128026 could regulate the expression of TNFAIP3-interacting protein 3 (*Tnip3*) and Dynamin-binding protein (*Dnmbp*), respectively, by competitively binding mmu-miR-6899-3p [5]. Nie, Zhao et al. explored the ceRNA network relationship between Lnc-ITSN1–2 and interleukin 23R (*IL-23R*), and elucidated their role in promoting CD4+ T cell activation, proliferation and Th1/Th17 cell differentiation [6].

Therefore, this study used the means of bioinformatics and machine learning to integrate the biopsy samples published in the Gene Expression Omnibus (GEO) public database to obtain differentially expressed genes (DEGs) related to the pathogenesis of UC. Screening of marker genes was carried out by means of machine learning and weighted gene co-expression network (WGCNA). Data sets from multiple countries were selected for validation to demonstrate that the differential expression of these marker genes is not an accidental result. Finally, the analysis of immune cell infiltration and the establishment of ceRNA network elucidate the potential mechanism of action of these marker genes affecting UC disease.

## Materials and methods

### Selection and download of the UC matrix dataset

The matrix files of normal human intestinal mucosal tissue and UC patient intestinal mucosal tissue samples were obtained from the GEO database (https://www.ncbi.nlm.nih.gov/geo/). The screening criteria are as follows: (1) *Homo sapiens* array expression profile; (2) Intestinal mucosal tissue biopsied from healthy people and UC patients; (3) The disease course is active; (4) The intestinal mucosal biopsy site is the colon; (5) The dataset contains more than 6 samples; (6) All included samples were not treated with drugs; (7) The dataset contains complete information about the sample. Finally, we selected two datasets for research: GSE75214 GPL6244 and GSE87466 GPL13158, with a total of 32 healthy human samples (control group) and 161 UC samples (treat group). In addition, the GSE37283 GPL13158, GSE134025 GPL13158, GSE160804 GPL20115, GSE38713 GPL570 and GSE179285 GPL6480 data sets were selected as the later validation data sets, including 47 healthy human colon samples and 48 UC colon samples, as shown in Table 1. Data from the GEO is publicly available and open to access. Therefore, no local ethics committee approval is required.

**Table 1:** Information for selected microarray datasets

GEO accession	Platform	Samples		Source tissue	Country	Attribute
		Con	UC			
GSE75214	GPL6244	11	74	colon	Belgium	Test set
GSE87466	GPL13158	21	87	colon	USA	Test set
GSE37283	GPL13158	5	4	colon	China	Validation set
GSE134025	GPL20115	3	3	colon		Validation set
GSE160804	GPL20115	3	3	colon		Validation set
GSE38713	GPL570	13	15	colon	Spain	Validation set
GSE179285	GPL6480	23	23	colon	USA	Validation set

**Fig. 1:**
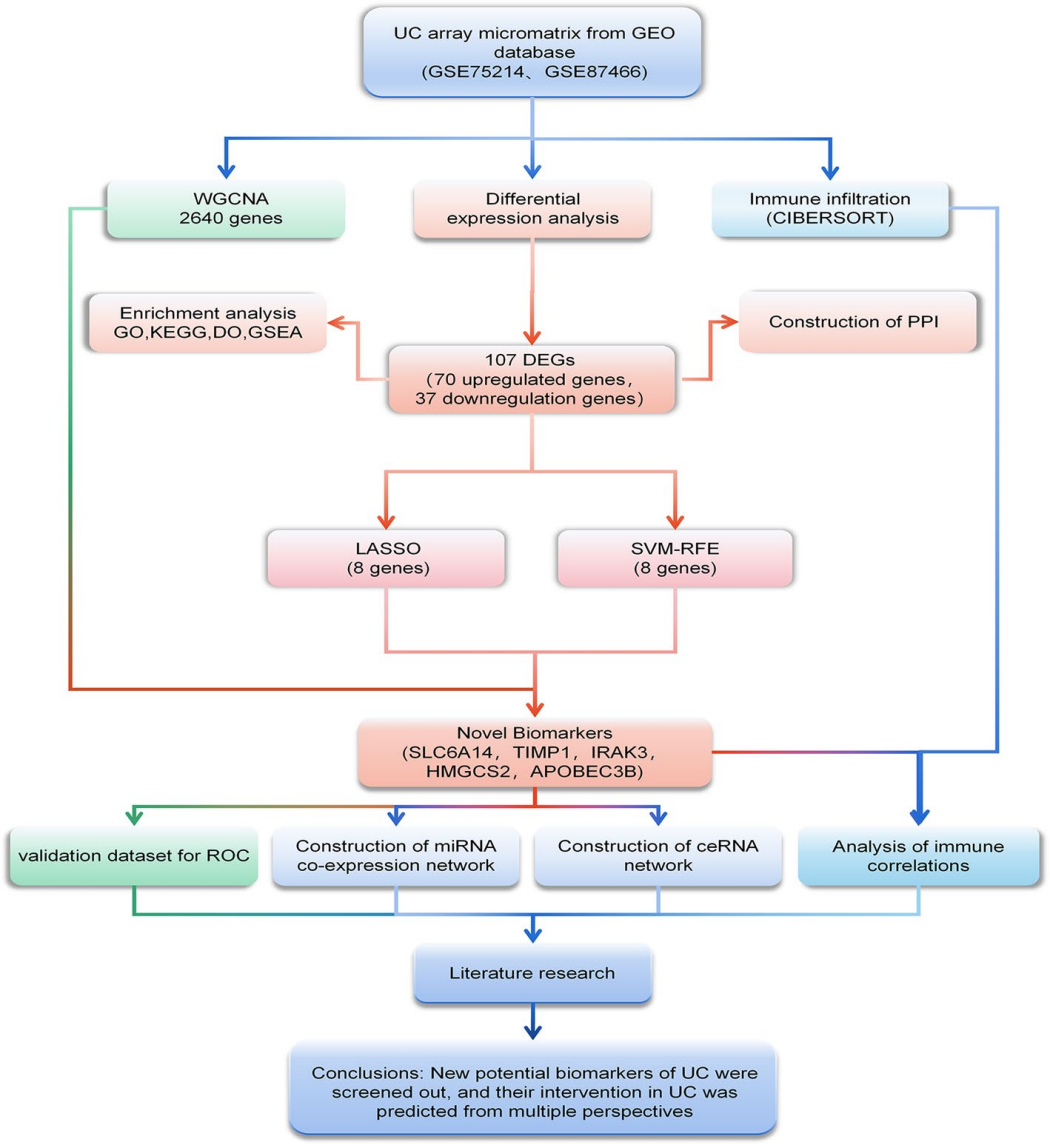
The flowchart of the analysis process

Con, Control group. *UC* Ulcerative colitis.

### Correction and screening of differentially expressed genes

The matrix files and platform files downloaded from the GEO database were organized and annotated using Perl language, and the matrix of probes was converted into the expression matrix of genes. The batch correction was performed using the ComBat function in the sva package in R language (version 4.1.2) to remove batch effects. DEGs were obtained by filtering the sample data using the R language limma package (http://www.bioconductor.org/packages/release/bioc/html/limma.html). The screening criterion was |LogFC| > 2, and the *p*-value was corrected by controlling the false discovery rate (FDR), taking the adjusted *p*-value (Q value) < 0.05.

### Visualization of differentially expressed genes

To more intuitively show which DEGs are up-regulated or down-regulated, the selected DEGs were visualized and analyzed, and heat maps and volcano maps were drawn.

### Construction of protein-protein interaction network

Import the DEGs into the String database (https://string-db.org/), select the *Homo sapiens* race, and obtain the protein-protein interaction network (PPI). Import PPI into Cytoscape 3.8.2 software for processing [7], and use the Minimal Common Oncology Data Elements (MCODE) tool in the software for cluster analysis (filtering criteria: degree cutoff = 2, node score cutoff = 0.2, k core = 2, maximum depth = 100) [8].

### Enrichment analysis

Various enrichment analyses were performed using the clusterProfiler package [9]. Gene ontology (GO) analysis can enrich the biological process (BP), cellular component (CC), and molecular function (MF) involved in these DEGs. The Kyoto Encyclopedia of Genes and Genomes (KEGG) analysis surveyed pathway enrichment. Disease Ontology (DO) analysis can identify diseases associated with these DEGs. Gene Set Enrichment Analysis (GSEA) evaluates the distribution trend of all genes and pathways in the sample based on the expression of all genes and pathways in the control and experimental groups and finds active genes and pathways to retain those expression changes in small but functionally important genes [10].

### Machine learning to screen disease genes

The obtained DEGs were further screened using machine learning methods to find genes associated with UC accuracy. Two machine learning algorithms, the Least absolute shrinkage and selection operator (LASSO) [11] and support vector machine-recursive feature elimination (SVM-RFE) [12], were used to screen disease potential biomarkers from DEGs. Weighted Gene Correlation Network Analysis (WGCNA) [13] enables significant association analysis of all genes to identify potential biomarkers or therapeutic targets.

### Data set to verify the characteristic expression genes of the disease

The GSE37283, GSE134025, GSE160804, GSE38713, and GSE179285 datasets were included as validation sets to verify whether the obtained UC signature genes also had significant differential expression in the validation set samples to prove that this was not an accidental result. Boxplots were drawn for validation analysis using the limma, ggplot2, and ggpubr packages in R.

### ROC curves of potential biomarkers in the test group and the validation group

Refers to the receiver operator characteristic curve (ROC), which is a comprehensive indicator that reflects the trade-off relationship between the sensitivity and specificity of continuous variables [14]. It can be used to verify the accuracy of the obtained genes as UC potential biomarkers. The area under the curve is infinitely close to 1, which means that the gene is more accurate as a disease potential biomarker. The ROC curves of the screened disease marker genes in the test group and the validation group were drawn respectively, so as to comprehensively determine the potential biomarker of the disease.

### Correlation analysis of potential biomarkers and immune cell infiltration

Using the R language, the correlation between UC and 22 types of immune cells was analyzed by the method of Cell-type identification by estimating relative subsets of RNA transcripts (CIBERSORT). Calculate the correlation coefficient and visualize the degree of immune cell infiltration, *p* value< 0.05. We chose to use the spearman coefficient to further study the correlation between marker genes and immune cells, to identify which immune cells they were significantly associated with, and to explore how marker genes play a role in UC by regulating immune cell infiltration.

### miRNA co-expression network and ceRNA network construction of signature genes

The ENCORI database (http://starbase.sysu.edu.cn/index.php) is a database [15] to study the interaction between RNAs, which integrates information from 7 public RNA databases such as TargetScan, microT, miRmap, and PITA. The database not only contains data based on predictions, but also provides experimental data support for co-immunoprecipitation, which is highly credible. By searching the ENCORI database and literature, the micro RNAs (miRNAs) of these five potential biomarkers were found for co-expression analysis, and appropriate miRNAs and long non-coding RNAs (lncRNAs) were selected according to the co-expression results to construct the ceRNA network.

## Research results

### Results of differentially expressed genes

**Fig. 2:**
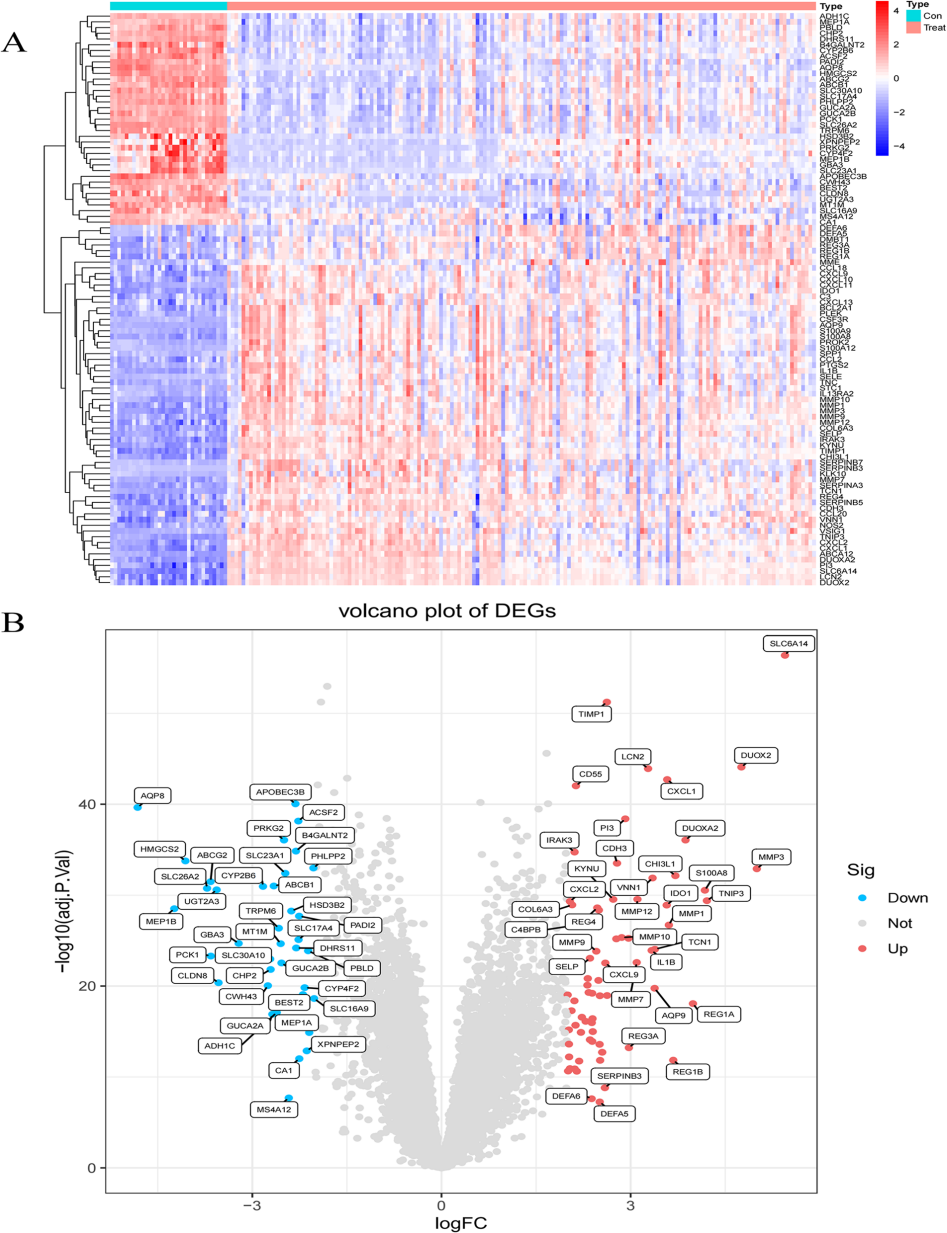
The heatmap and a volcano plots. **A** The heatmap of DEGs distribution in GSE75214 and GSE87466; **B** The volcano plots of DEGs. Red represented a high expression of DEG, while blue represented a low expression of DEG.

Fig. 1 Illustrates the workflow of this study. We included 32 healthy human intestinal mucosal biopsy samples (con group) and 161 UC patients’ active colonic mucosal tissue samples (treat group) from the GSE75214 and GSE87466 datasets. A total of 107 DEGs were screened, including 70 up-regulated genes and 37 down-regulated genes. (Fig. 2A-B)

### PPI network analysis and MCODE cluster modules

**Fig. 3:**
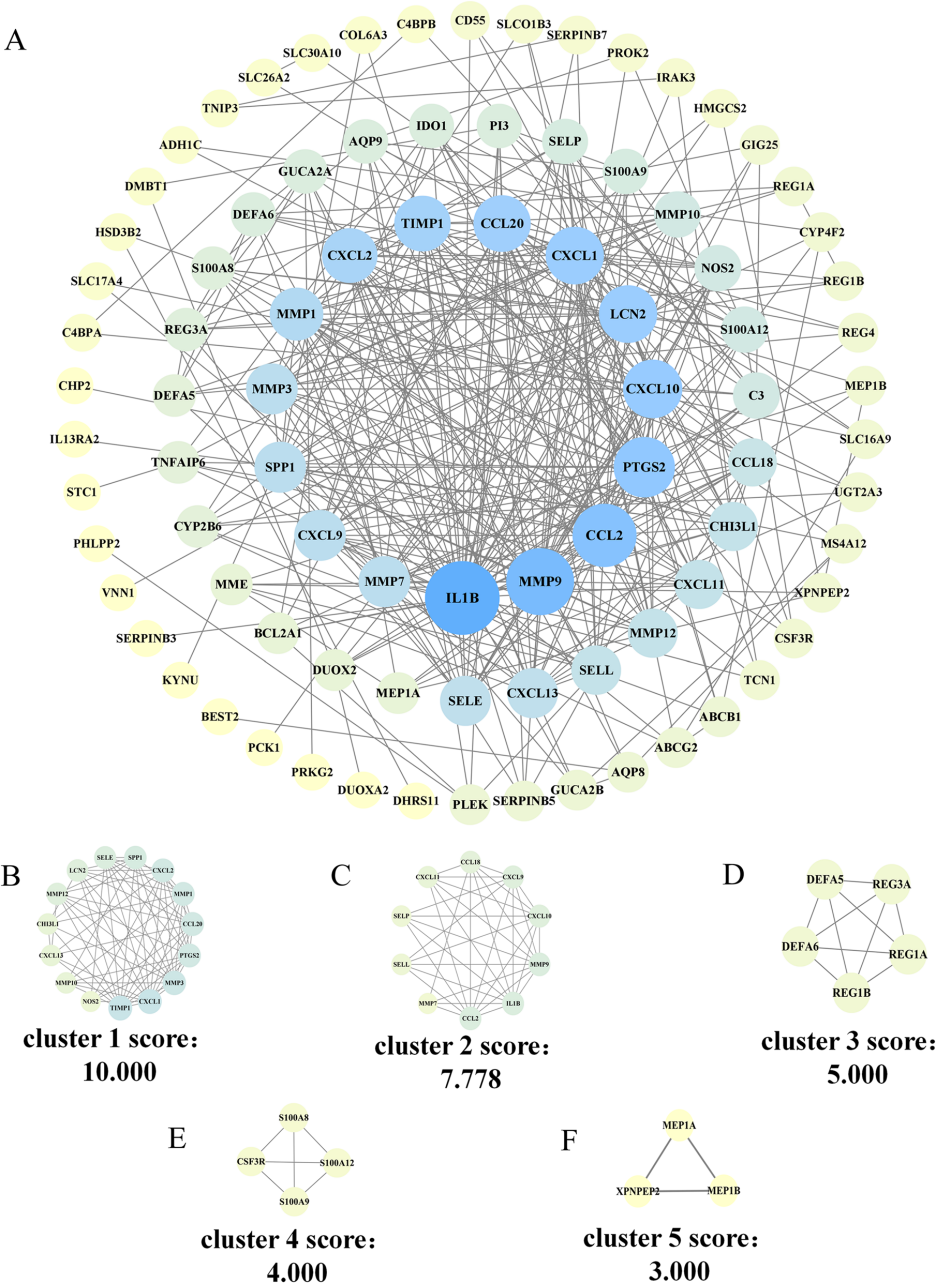
PPI network of DEGs and five cluster modules extracted by MCODE. **A** The interaction network between proteins coded by DEGs was comprised of 107 nodes and 370 edges. The closer the color is to blue, the more complex the relationship between the proteins in the network is, while the yellower the color, the simpler the relationship. **B-E** Five cluster modules were extracted by MCODE

The 107 DEGs obtained by screening were imported into the String database to obtain the PPI network. Among them, cytoplasmic β-glucosidase (*GBA3*) could not be identified by the String database, so after querying the uniport database, it was replaced with the synonymous name *CBG* of this target. Finally we obtain a PPI network composed of 107 nodes and 370 edges. The average node degree of this network is 6.92, (Fig. 3A). In this network, we identified five modules based on filtering criteria. Cluster 1 had the highest cluster score (score 10.000, 15 nodes, 70 edges), Cluster 2 (score 7.778, 10 nodes, 35 edges), Cluster 3 (score 5.000, 5 nodes, 10 edges), cluster 4 (score 4.000, 4 nodes, 6 edges) and cluster 5 (score 3.000, 3 nodes, 3 edges), and cluster 5 (score 3.000, 3 nodes, 3 edges) (Fig. 3B-F).

### Enrichment analysis

**Fig. 4:**
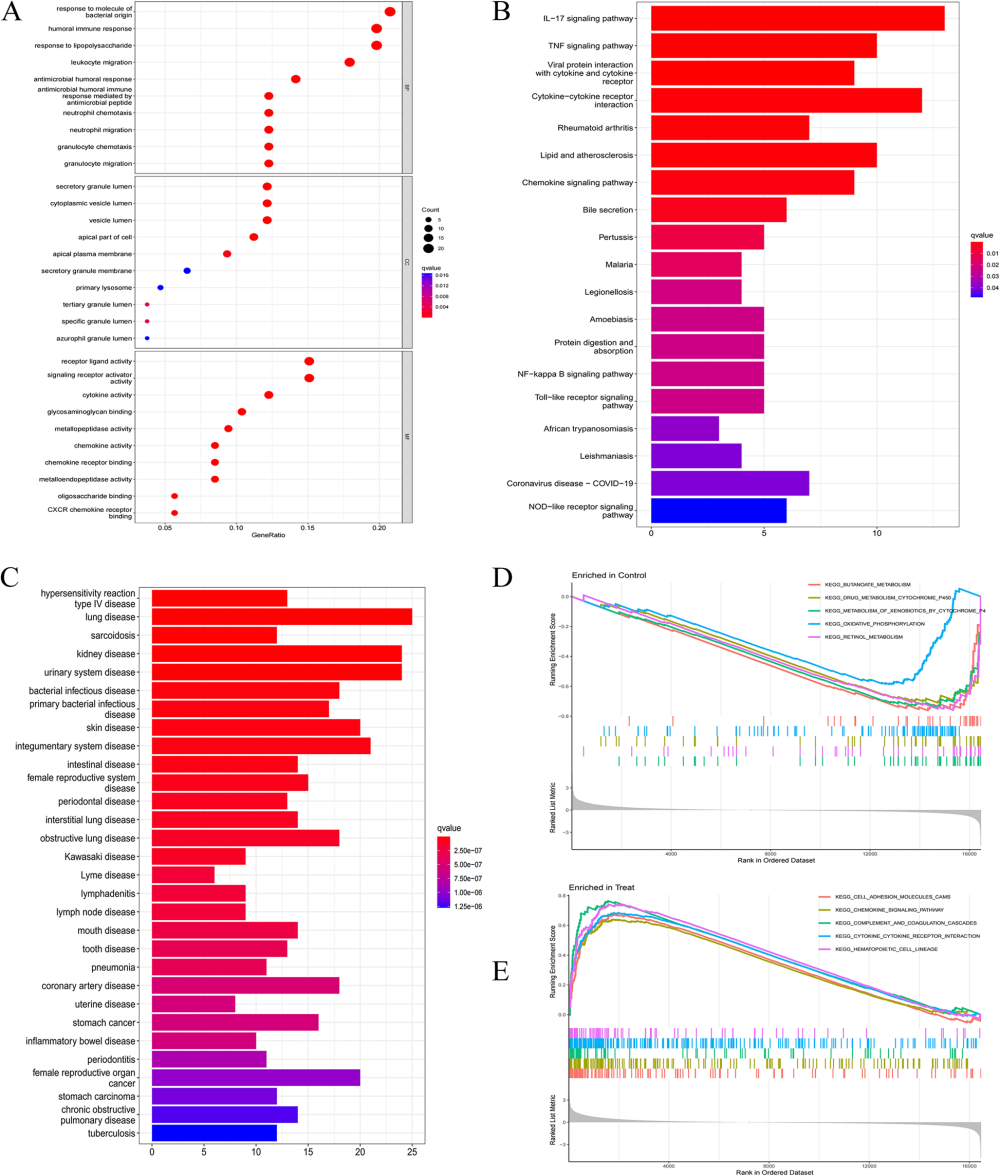
GO, KEGG pathway, DO, and GSEA enrichment analyses of DEGs. **A** The bubble plot shows the top 10 enriched results of DEGs from three aspects of BP, CC, and MF; **B** The barplot shows the most enriched KEGG pathways of DEGs; **C** The barplot of DO enrichment analysis; **D-E** GSEA plots depicting the five significant genes or pathways

GO enrichment analysis showed that DEGs in BP were mainly involved in the body’s response to molecules of bacterial origin, humoral immune response, antibacterial humoral response, and response to lipopolysaccharide. In addition, it also has a significant impact on the involvement of neutrophils in mediating immune responses. In terms of MF, DEGs are mainly involved in receptor-ligand activity, activation of signaling receptor activators, activation of cytokines, and binding of glycosaminoglycans. Then, in terms of CC, it was shown that DEGs were mainly distributed in the secretory granule lumen, cytoplasmic vesicle lumen, and vesicle lumen (Fig. 4A). KEGG pathway enrichment analysis showed that DEGs were enriched in the IL-17 pathway, tumor necrosis factor α (*TNF-α*) pathway, NF-κB signaling pathway, and Toll-like receptor signaling pathway (Fig. 4B). The DO enrichment analysis can be seen in Fig. 4(C), from which diseases related to these DEGs can be found, such as hypersensitivity reaction type IV disease, lung disease, and sarcoidosis. This provides support for finding interactions between active UC and other diseases.

In the GSEA enrichment analysis, we found 5 genes or pathways that were most significantly enriched between the con group and the treat group. The results were cell adhesion molecules (CAMs), chemokine signaling Pathway, complement and coagulation cascades, cytokine-cytokine receptor interaction, and Hematopoietic cell lineage (Fig. 4D-E).

### Machine learning to screen potential biomarkers

**Fig. 5:**
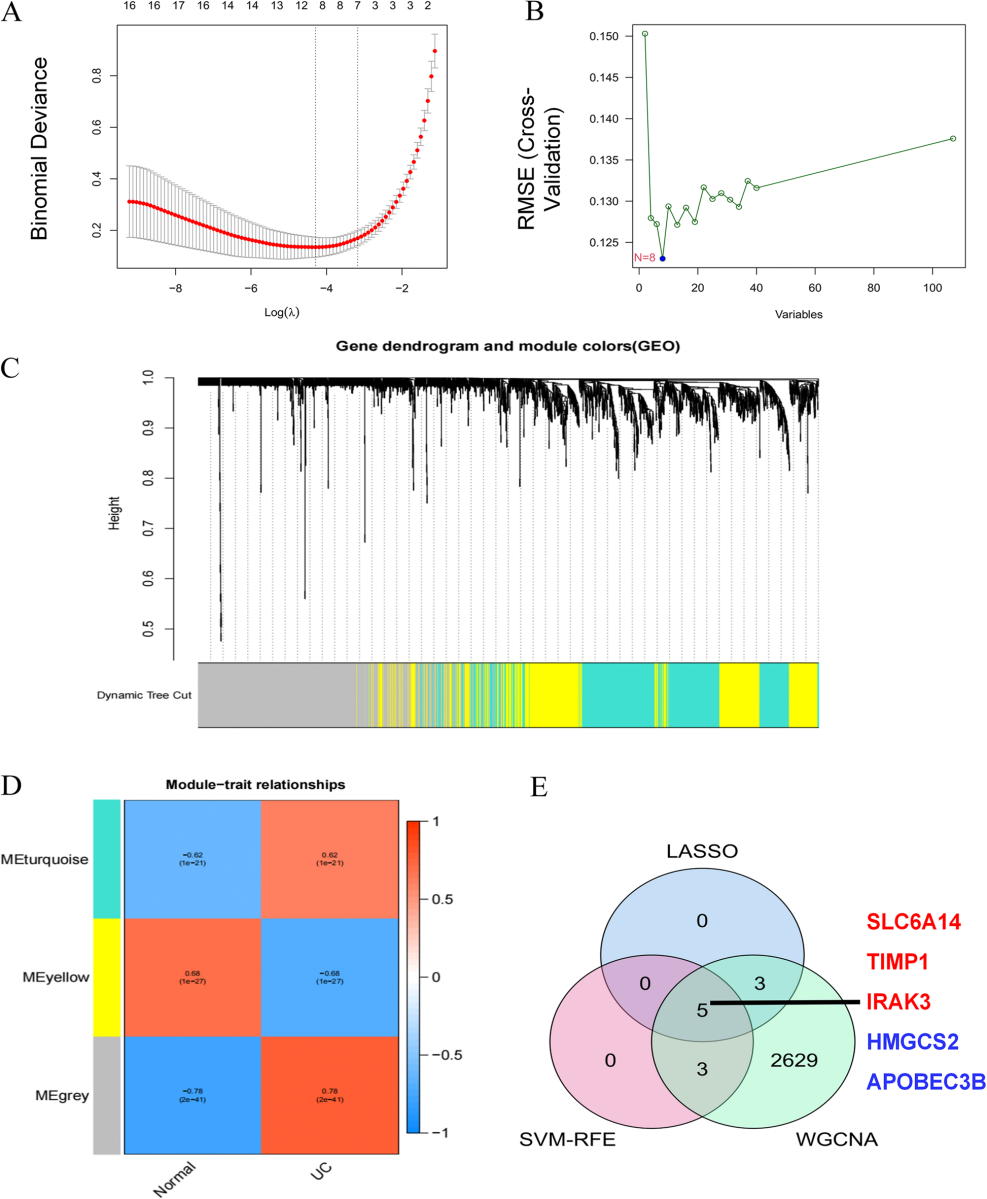
Screening of potential biomarkers by machine learning. **A** LASSO logistic regression algorithm to screen disease potential biomarkers; **B** Based on SVM-RFE algorithm to screen disease potential biomarkers; **C** UC module clustering dendrogram based on a dissimilarity measure (1-TOM); **D** Heatmap of the correlation between module eigengenes and active UC; **E** The intersection of the results of the two algorithms and WGCNA analysis

The LASSO logistic regression algorithm and the SVM-RFE algorithm each identified 8 genes that could be used as biomarkers for UC (Fig. 5A-B). WGCNA analysis divides genes into different modules with similar biological functions. In a total of 6 key modules, 2640 genes were identified that were significantly associated with UC (Fig. 5C-D). To improve the accuracy of machine learning screening results, 5 genes identified under the two algorithms and WGCNA analysis were selected as disease signature genes. They are sodium- and chloride-dependent neutral and basic amino acid transporter B (*SLC6A14*), metalloproteinase inhibitor (*TIMP1*), DNA dC- > dU editing enzyme (*APOBEC3B*), interleukin-1 receptor-associated kinase 3 (*IRAK3*) and hydroxymethylglutaryl-CoA synthase (*HMGCS2*) (Fig. 5E).

### Validation of disease signature expressed genes

**Fig. 6:**
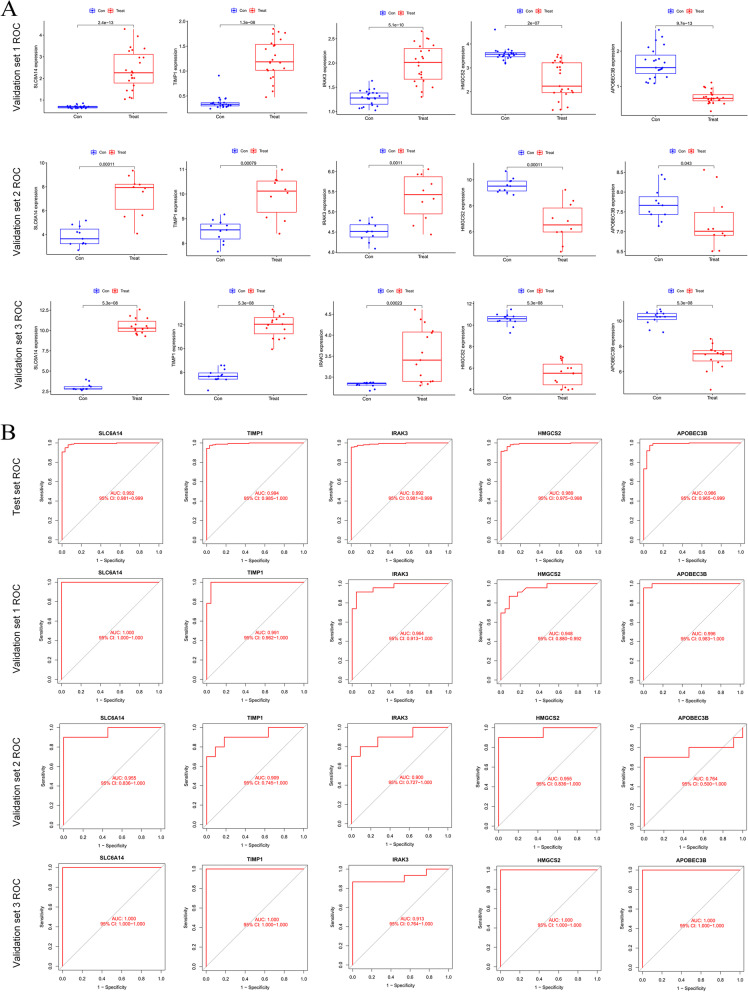
Expression of potential biomarkers and ROC validation. **A** Expression of marker genes in the intestinal mucosa of healthy and active UC patients in the validation dataset. Blue represents the con group, and red represents the treat group; **B** ROC curve plot of marker genes in test set and validation set. Validation set 1 is the result from a merged dataset of GSE37283, GSE160804, and GSE134025, validation set 2 is the result from GSE38713, and dataset 3 is the result from the GSE179285 dataset

In order to verify the reliability of our screening results, we selected datasets from the Americas, Europe and Asia for validation, which are Validation set 1: a merged dataset of three small-sample datasets from China (GSE37283, GSE134025, GSE160804); Validation set 2: a dataset from Spain (GSE38713); Validation set 3: a dataset from the United States (GSE179285), see Table 1. Boxplots were drawn to show the expression results of these eigengenes in different datasets. The results showed that these five marker genes were significantly differentially expressed in the validation set (*p* value< 0.05). *SLC6A14*, *TIMP1*, and *IRAK3* were up-regulated in the validation set, while *HMGCS2* and *APOBEC3B* were down-regulated. This is consistent with the conclusions obtained in the test set (Fig. 6A). Both ROC curves and AUC indicated that these 5 potential UC biomarkers had high confidence in both test and validation sets (Fig. 6B).

### Infiltration of immune cells results

**Fig. 7:**
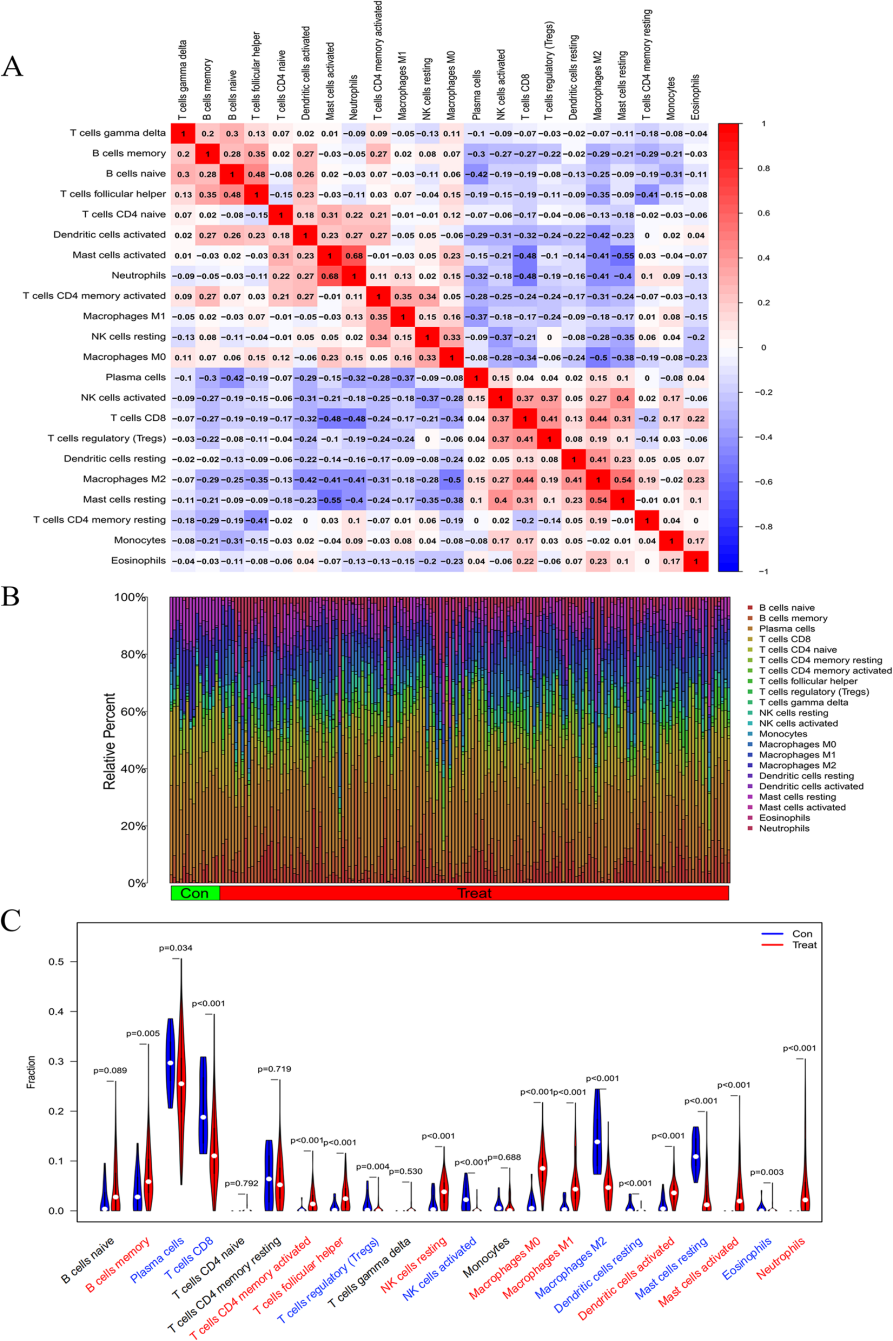
UC immune cell infiltration results. **A** Heatmap of correlation in 22 types of immune cells. Red represents a positive correlation, and blue represents a negative correlation. Darker color implies stronger association.; **B** Barplot of the proportion of 22 types of immune cells; **C** Vioplot for immune cell infiltration analysis. Blue represents decreased infiltration of this type of immune cells, red represents increased infiltration and black represents insignificant differential infiltration

Using the CIBERSORT algorithm, a summary analysis was first performed on 32 normal human samples and 161 UC patient biopsy samples in the test set. Through the correlation heat map, it can be seen that among the 22 immune cells, neutrophils and activated mast cells, follicular helper T cells and naive B cells, M2 macrophages, and resting mast cells were significantly positively correlated. There was a significant negative correlation between resting mast cells and activated mast cells, T cell CD4 and activated mast cells, and neutrophils and T cell CD4 (Fig. 7A). Compared with normal samples, the distribution of immune cells in the intestinal mucosa of UC patients changed significantly, the infiltration of neutrophils significantly increased, the M0 and M1 macrophages were relatively increased, and the M2 macrophages were relatively decreased (Fig. 7B). Seventeen types of immune cells were significantly different between the two groups by drawing a violin plot (*p* < 0.05). Among them, the infiltration of neutrophils, M0 macrophages, M1 macrophages, activated Dendritic cells (DC), and activated memory T cells CD4 was significantly increased. While Infiltration of regulatory T cells (Tregs), M2 macrophages, activated mast cells, and resting DC cells was significantly reduced (Fig. 7C).

### Correlation analysis between disease potential biomarkers and immune cells

**Fig. 8:**
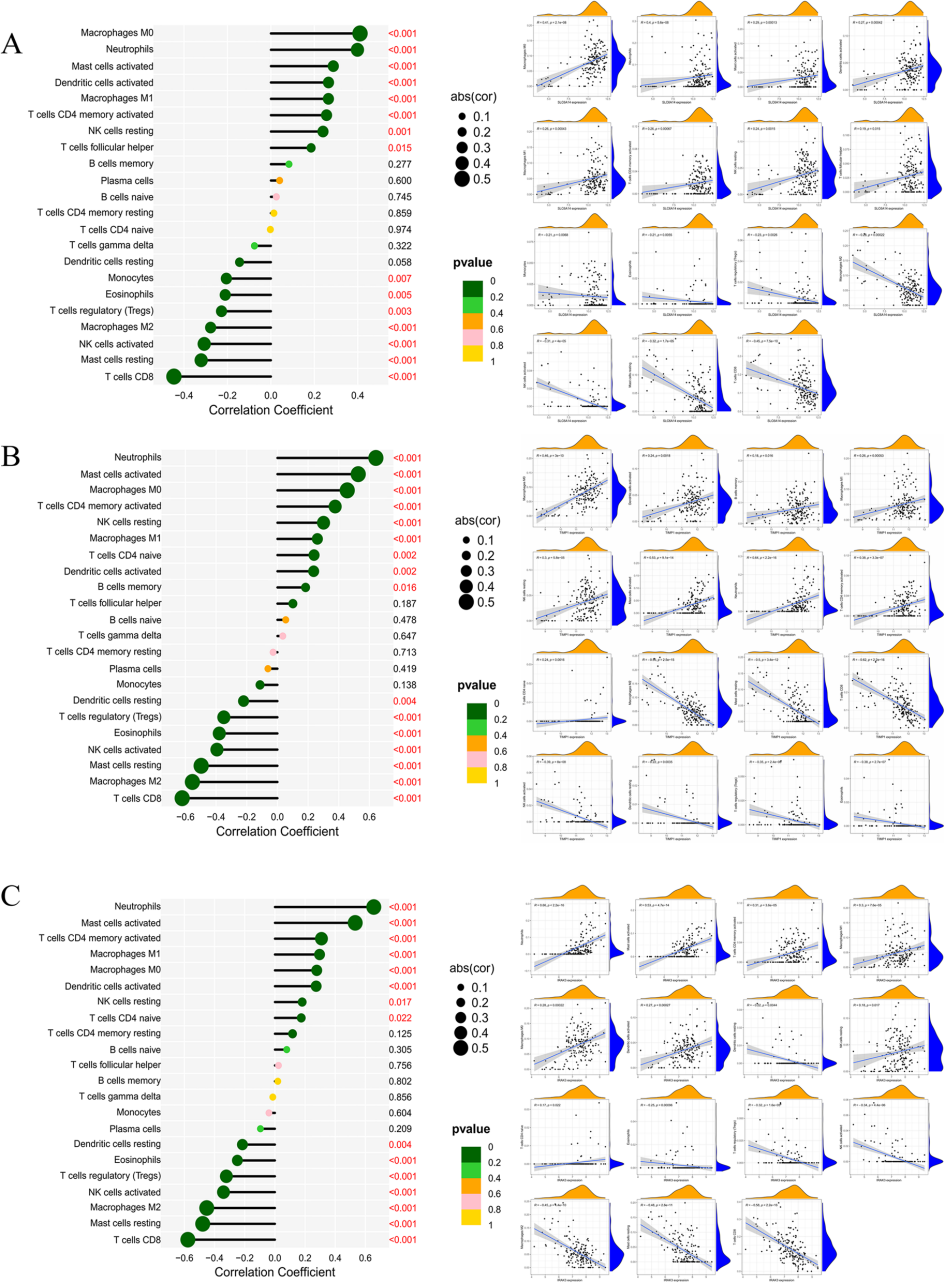
Immune cell correlation analysis panel of up-regulated marker genes. **A** Lollipop and scatter plots of the correlation between *SLC6A14* and immune cells; **B** Lollipop and scatter plots of the correlation between *TIMP1* and immune cells; **C** Lollipop and scatter plots of the correlation between *IRAK3* and immune cells

**Fig. 9:**
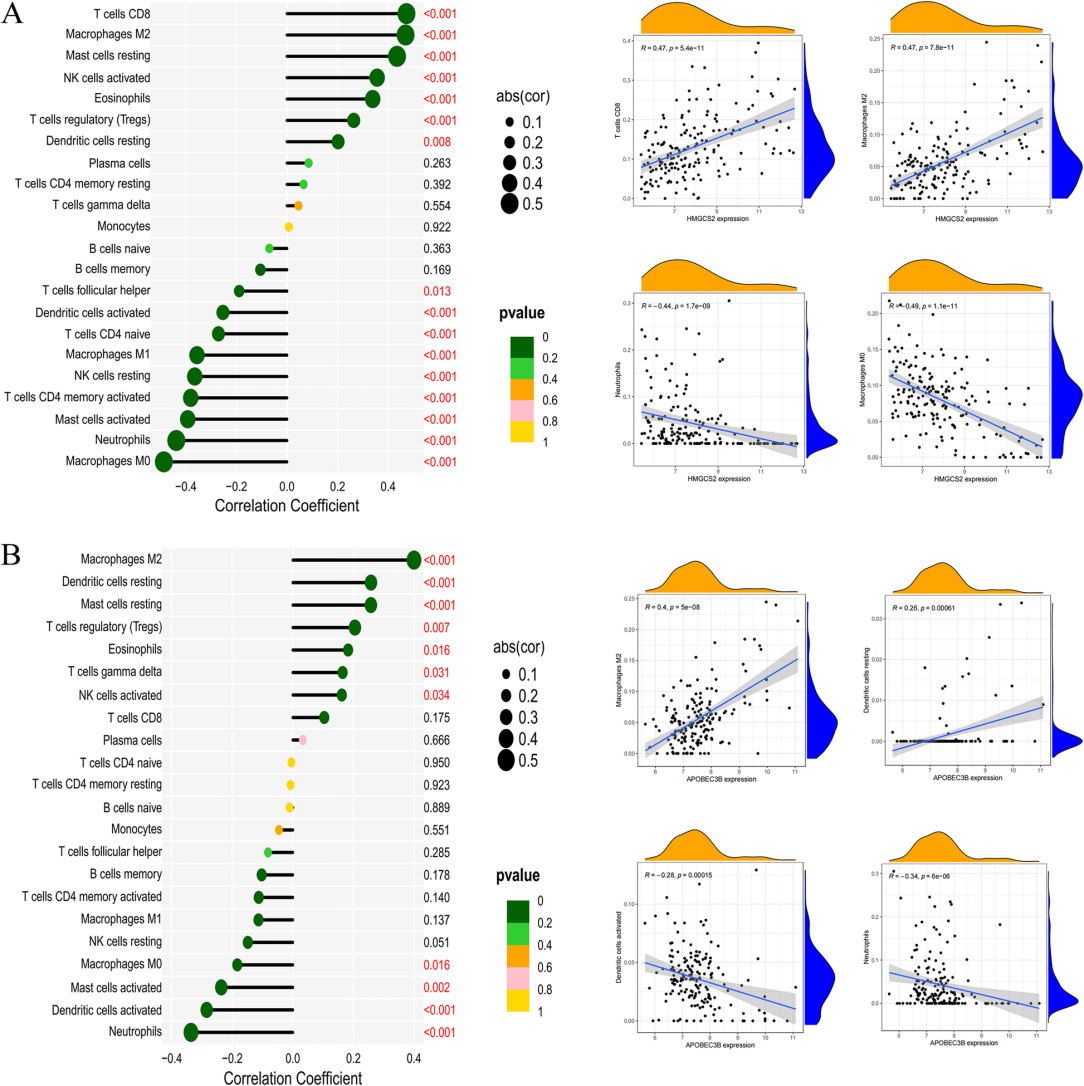
Immune cell correlation analysis panel of down-regulated marker genes. **A** Lollipop and scatter plots of the correlation between *HMGCS2* and immune cells; **B** Lollipop and scatter plots of the correlation between *APOBEC3B* and immune cells

The correlation analysis of marker genes and 22 kinds of immune cells can help us speculate how these genes participate in the course of UC by regulating the infiltration of immune cells. The up-regulated marker genes *SLC6A14*, *IRAK3,* and *TIMP1* in UC are positively correlated with neutrophils, activated mast cells, activated memory T cells, M0 and M1 macrophages; with T cells CD8, resting mast cells, activated NK cells, M2 macrophages, and regulatory T cells (Tregs) were negatively correlated (Fig. 8A-C). The down-regulated *HMGCS2* and *APOBEC3B* in UC were positively correlated with M2 macrophages, T cell CD8, resting DC cells, resting mast cells, activated NK cells, and regulatory T cells (Tregs); and neutrophils, M0 macrophages, activated mast cells, activated dendritic cells, memory T cells CD4 were negatively correlated (Fig. 9A-B).

### Construction of miRNA co-expression network and ceRNA network of potential biomarkers

**Fig. 10:**
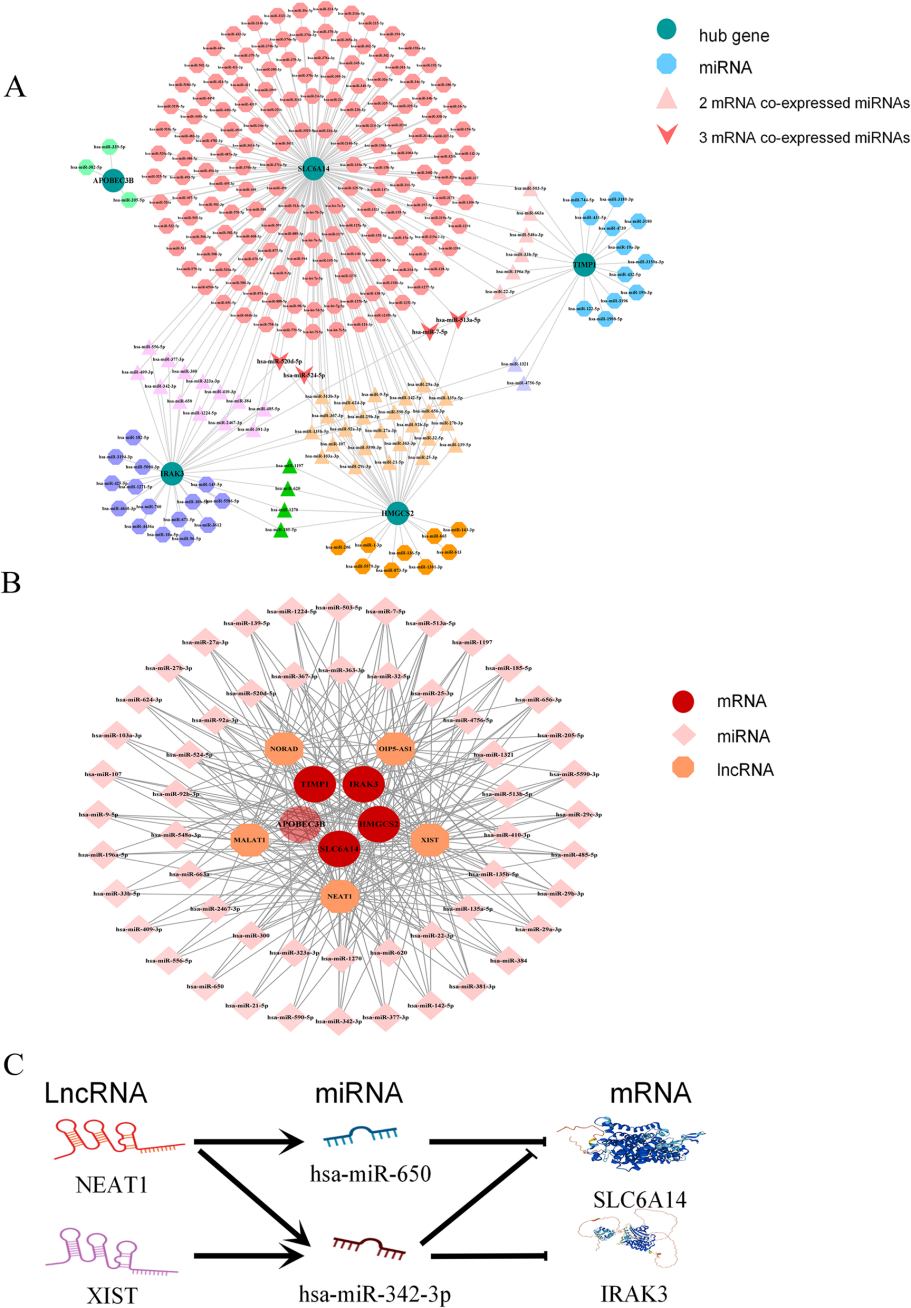
The miRNA co-expression network and ceRNA network of marker genes. **A** The miRNA co-expression network of five signatures; **B** The ceRNA network of mRNA-miRNA-lncRNA; **C** Schematic diagram of novel regulatory pathways in the pathogenesis of UC

The ENCORI database was used to search for miRNAs related to marker genes. The mRNA-miRNA results were as follows: 202 related to *SLC6A14*, 22 related to *TIMP1*, 37 related to *IRAK3*, 40 related to *HMGCS2,* and 3 related to *APOBEC3B*. The results were drawn into a co-expression network by Cytoscape software, and the co-expression relationship between miRNA and mRNA was marked with different graphs (Fig. 10A). In the miRNA co-expression network, we found 54 target miRNAs with broad correlations among the marker genes (S1 Table). Taking these miRNAs as the object of our further research, we imported them into the ENCORI database to search for lncRNAs that interacted with them. The screening criteria were: mammalian, human h19 genome, strict stringency (> = 5) of CLIP-Data, with or without degradome data and successful retrieval in at least two public databases. Ultimately, we selected lncRNAs that were prevalent in most miRNAs prediction results for inclusion in our study. Ultimately, we selected lncRNAs that were prevalent in most miRNAs prediction results for inclusion in our study. They are non-coding RNA activated by DNA damage (NORAD), OIP5 antisense RNA1 (OIP5-AS1), X inactive specific transcript (XIST), metastasis-associated lung adenocarcinoma transcript 1 (MALAT1), and nuclear paraspeckle assembly transcript 1 (NEAT1). According to the ceRNA hypothesis, the screened miRNAs, lncRNAs, and mRNAs were constructed into a network (S2 Table and Fig. 10B).

Through the node statistics of the network, we found that NEAT1 and XIST were lncRNAs that were closely related to these five disease marker genes. Then, we further performed a literature search and selected 2 miRNAs (miR-342-3p/miR-650) and 2 lncRNAs (NEAT1/XIST) that with related reports in UC or UC-related colorectal cancer. We make a bold inference that NEAT1/miR-342-3p-*SLC6A14*, NEAT1/miR-650/*SLC6A14*, NEAT1/miR-650/*IRAK3*, XIST/miR-342-3p/*IRAK3* may serve as the novel regulatory pathway in the pathogenesis of UC (Fig. 10C).

## Discussion

Ulcerative colitis is a chronic non-specific intestinal inflammatory disease. Most patients have a slow onset, and the severity of the disease varies. In the early onset, the main manifestations are abdominal pain, diarrhea, mucus stool, and bloody stool. The clinical diagnosis is mainly colonoscopy. Since the etiology and pathogenesis of the disease are not yet clear, clinical diagnosis and treatment methods are still based on dynamic monitoring of objective inflammation and symptomatic treatment. The search for new potential biomarkers is of great significance in deepening the research on the pathogenesis of UC, optimizing the diagnosis and treatment methods, and developing new drugs.

According to bioinformatics analysis, we screened 107 DEGs in 193 samples. In the enrichment analysis, it was found that these DEGs were closely related to the body’s humoral immune function, inflammatory response, and antibacterial humoral response. In the enrichment analysis, it was found that these DEGs were closely related to the body’s humoral immune function, inflammatory response, and antibacterial humoral response. GSEA enrichment analysis additionally revealed that these DEGs were most closely associated with CAMs, chemokine signaling pathways, complement and coagulation cascades, cytokine-cytokine receptor interactions, and hematopoietic cell lineage pathways.

CAMs are glycoproteins that play key roles in biological processes such as hemostasis, immune response, and inflammation [16]. For example, leukocyte-binding mucosal addressin cell adhesion molecule (*MADCAM*), its blockade can attenuate the transfer of lymphocytes to the intestinal mucosa of UC patients [17, 18]. Chemokines induce cell-directed chemotaxis that recruits leukocytes to critical sites of inflammation after local injury [19], such as C-C motif chemokine ligand 20 (*CCL20*). *CCL20* can be induced by pro-inflammatory signals such as *TNF-α* or *TLR*, bind *CCR6* and induce the recruitment of B cells with high *CCL6* expression into intestinal epithelial cells of patients with inflammatory bowel disease in response to inflammatory stimuli [20–22]. Complement is an important mediator in the innate immune response, involved in the recruitment of inflammatory and immune-competent cells, and is of great significance for the detection, regulation, and elimination of foreign pathogens as well as self-apoptotic or malignant cells [23]. Sünderhauf et al. observed that when intestinal mucosal injury and inflammation are active in IBD patients, complement *C3* expression is increased, and local *C3a* production is increased, that in turn propagates pro-in-flammatory cytokine secretion by innate lymphocytes [24]. Thrombin generated during blood coagulation can activate members of G protein-coupled receptors such as protease-activated receptors (*PAR*), which can mediate the process of the innate immune system, thereby affecting the inflammatory response [25]. Cytokines are proteins that participate in biological processes such as innate and adaptive inflammatory responses, host defense, and so on, and aim to restore the balance of the microenvironment in the body. The interleukin family, tumor necrosis factor *TNF-α*, interferon, etc. it contains are considered to be the key pathways regulating the progression of IBD disease [26, 27]. Biologics made based on *TNF-α*, such as Infliximab and Adalimumab, are widely used in the induction and maintenance of remission in IBD. Hematopoietic stem cells differentiate into leukocytes and their lymphoid lineages—NK cells, T lymphocytes, and B lymphocytes, which affect immune and inflammatory processes [28].

It is well known that immune homeostasis depends on immune cells and immune molecules. Innate immune cells such as M1 macrophages, NK cells, immunogenic DC cells, and adaptive immune cells such as CD4+ and CD8+ cells play a role in promoting mucosal immune and inflammatory responses in UC [29–31]; While M2 macrophages, regulatory NK cells, regulatory DC cells in innate immune cells, and Tregs cells in adaptive immune cells can release a variety of anti-inflammatory factors to play an antagonistic role in reducing intestinal damage in UC [32–34]. After immune cell correlation analysis of novel marker genes, we found that *SLC6A14*, *TIMP1*, and *IRAK3*, whose expression were up-regulated in UC, also increased the pro-immune and pro-inflammatory cells they affected; While the down-regulated expression of *HMGCS2* and *ABOPEC3B* also reduced the proportion of immune cells with the anti-immune response and anti-inflammatory effects. Such results provide immunological support for their role in UC development.

Further review of relevant literature and research, we found that these five potential biomarkers play an important role in biological processes such as tumor development and metastasis, the immune function of the body, the development of inflammation, and maintenance of the intestinal barrier. However, there is not much research and discussion on them and IBD by researchers.

As a sodium (Na^+^) and chloride (Cl^−^) dependent amino acid transporter, *SLC6A14* is mainly involved in the transmembrane transport of amino acids and Na^+^-Cl^−^ [35, 36]. In addition to playing an important nutritional support role in the development of cancer [37], *SLC6A14* also effectively antagonizes toxins produced by a variety of infectious bacteria, and improves the reabsorption of Na + by intestinal cells, relieves diarrhea symptoms, and maintains homeostasis [38]. The expression changes of *SLC6A14* can suggest the pathogenic ability of gut microbes in UC and can be used to monitor the changes in the microbial community in the course of UC [39, 40]. This suggests that the index of *SLC6A14* can provide certain guidelines for the use of antibiotics and probiotics during the treatment of IBD.

Carcinogenesis is one of the most serious complications of UC, and long-term UC has a higher risk of progression to CRC [41]. *HMGCS2* is an enzyme involved in catalyzing ketosis in mitochondria, which not only determines the ketogenic ability of the colon, but also provides lipid-derived energy for tumor cells, and affects tumor development and migration [40]. It can also synergize with butyrate to promote mitochondrial oxidation, enhance oxidative stress response [42], and inhibit human endothelial cell growth and angiogenesis [43]. The increase of ketogenic effect can reduce the accumulation of immunosuppressive cells in the tumor, increase the infiltration of NK cells and cytotoxic T cells, and enhance the anticancer effect of PD-1 blockade in CRC [44]. Studies have found that *HMGCS2* is significantly up-regulated in the intestinal mucosa of long-term UC patients [40], but down-regulated in most colorectal tumors [45]. Some researchers believe that *HMGCS2* can be used as a monitoring indicator for the prognosis of colorectal cancer (CRC) and CRC radiotherapy and chemotherapy [44, 46]. But whether there is a closer link between this differential expression and the carcinogenesis of UC remains unclear.

As one of the metalloproteinase inhibitors, *TIMP1* can combine with matrix metalloproteinases (*MMPs*) such as *MMP10* and *MMP13* to form irreversible complexes to inhibit the synthesis and secretion of proteases and reduce the destruction of collagen. It plays an important role in maintaining the intestinal barrier and regulating tumor progression [47, 48]. Compared with healthy people, there may be two distinct expressions of *TIMP1* in the blood of UC patients: low expression due to insufficient activity or concomitant increase in the regulation of matrix metalloproteinases [49, 50]. Colitis mouse studies also showed that the expression of *TIMP1* was increased during the active period of inflammation and decreased significantly during the recovery period [51]. This is consistent with our findings. It can be seen in Fig. 3 that in addition to *TIMP1*, *MMP1*, *MMP3*, *MMP7*, and *MMP9* in the matrix metalloproteinase family were significantly expressed in the intestinal mucosa of UC patients. This may be related to the fact that *TIMP 1* antagonizes the overexpression of *MMPs* in UC and inhibits the involvement of *MMPs* in the shaping of the inflammatory microenvironment in UC [52]. In terms of immune cells, Wu believes that *TIMP1* is a key gene involved in the infiltration of immune cells in thyroid cancer lymphatic metastasis [53]. *TIMP1* potently inhibits the polarization of NK cells toward a decidual-like phenotype [54] and increases hepatic neutrophil infiltration [55]. In addition, *TIMP1* has also been shown to play a role in the prognosis of IBD-related CRC [56]. In conclusion, we speculate that *TIMP1* can be used to monitor the healing of the intestinal mucosal barrier in UC patients.

As the endogenous source of somatic mutations in various cancers, the APOBEC family participates in and affects the immune response of the body [57]. *APOBEC3B* is not only regulated by *TNF-α* to affect the evolution of cancer cells in the inflammatory microenvironment [58] but also acts as a DNA dehydrogenase to effectively inhibit retroviral replication and retrotransposon migration. Thereby, it has a defensive effect on the virus, promotes DNA demethylation, and participates in the body’s innate immune response and the conversion of cytidine to uridine [59–61]. Wang found that *APOBEC3B* can promote the growth of liver tumor cells through the NF-κB signaling pathway, promote the recruitment of tumor macrophages, and increase the CD8 + T-expressing myeloid-derived suppressor cells and PD1 [62]. Therefore, whether *APOBEC3B* is specifically expressed in IBD and related CRC remains to be studied.

*IRAK3* (or *IRAK-M*) is closely related to *IL1R*, Toll receptor signaling, and lipopolysaccharide signaling [63]. *IRAK3* can inhibit the dissociation of *IRAK* families 1 and 4 under the induction of Toll-like receptors and can act as a negative feedback regulator to intervene in Toll-like receptor signaling and innate immune homeostasis and regulate inflammatory responses [64–66]. It controls the inflammatory response magnitude of macrophages to TLR signaling, inhibits lipopolysaccharide-induced NF-κB activation in macrophages, and reduces NK cell abundance [67–70]. In addition, *IRAK3* can induce DC cells through *IL33* to upregulate the expression of inflammatory factors such as *IL6*, and increase the inflammatory response [71]. Zhang et al. found that *IRAK3*-deficient neutrophils can enhance the ability of effector T cell proliferation and activation, effectively enhancing anti-tumor immune responses [72]. The study found that *IRAK3* was significantly up-regulated in the intestinal mucosa of UC inactive stage, while the expression of *IRAK3* in the remission stage was similar to that of healthy people [73]. We believe that *IRAK3* can be used to monitor objective inflammation and to assist in detecting the degree of inflammatory changes, and its close relationship with the *TLR* receptor signaling pathway deserves more in-depth study.

After obtaining these potential biomarkers, we also predicted their related miRNAs and lncRNAs through the database and searched for the relationship between them through a literature search. Ultimately, we focused our attention on the marker genes *SLC6A14* and *IRAK3*, lncRNAs NEAT1 and XIST, miR-342-3p, and miR-650. It has been reported that knockdown of XIST can indirectly reduce the expression of transforming protein RhoA (*RhoA*) at the mRNA and protein levels through the ceRNA relationship, thereby improving the development of inflammatory CRC [74]. NEAT1 is up-regulated in the intestinal mucosa of UC and can affect the development of UC by affecting *TNF*-related receptors [75]. Studies have shown that the expression of miR-342-3p is decreased in the sigmoid colon region of UC patients [76], but blocking NEAT1 can improve the expression of miR-342-3p, thereby reducing cellular inflammation and lipid uptake [77]. In addition, miR-650 was also shown to act as an upstream regulator of the LRR and PYD domains-containing protein 6 (*NLRP6*). After being overexpressed, miR-650 can effectively inhibit *NLRP6* and reduce the inflammatory response and apoptosis of UC [78]. Through literature research and bioinformatics predictions, we propose a bold hypothesis that NEAT1/miR342-3p/*SLC6A14*、NEAT1/miR-650/*SLC6A14*、NEAT1/miR-650/*IRAK3*、XIST/miR-342-3p/*IRAK3* ceRNA relationship axis plays an important role in the occurrence and development of UC. Unfortunately, at present, the experimental research and related drug development of these 5 potential biomarkers are very scarce, and it is difficult to combine clinical data and experiments to explore more deeply, which makes our hypothesis lack of strong support. In future studies, we will experimentally validate the findings in vitro and in vivo. It is also necessary to propose effective strategies for in-depth clinical validation, such as increasing follow-up time to validate the results, using methods such as multiple regression modeling to validate and improve the specificity and sensitivity of biological markers, and so on.

## Conclusion

Our work identifies five UC potential biomarkers: *SLC6A14*, *TIMP1*, *IRAK3*, *HMGCS2*, and *APOBEC3B* as potential biomarkers for UC diagnosis and treatment, and boldly predicts their mechanisms of action at the immune cell infiltration and transcriptome levels. Furthermore, based on the screening results, we propose that NEAT1/miR-342-3p-*SLC6A14*、NEAT1/miR-650/*SLC6A14*、NEAT1/miR-650/*IRAK3*、XIST/miR-342-3p/*IRAK3* may serve as a potential RNA regulatory pathway to monitor and control UC progression.

## Supplementary Information

**Additional file 1.** The Supplementary Material for this article can be found online at Mendeley Data, V1, DOI:https://doi.org/10.17632/58hf8kz9tm.1

## Data Availability

The code and raw data used in the research have been uploaded to the Github database, https://github.com/782678245/Screening-of-ulcerative-colitis-biomarkers.git

## Abbreviations

UC: Ulcerative colitis
CD: Crohn’s disease
IBD: Inflammatory bowel diseases
GEO: Gene Expression Omnibus
DEGs: Differentially expressed genes
ceRNA: Competing endogenous RNA
FDR: Controlling False Discovery Rate
PPI: Protein protein interaction network
GO: Gene ontology analysis
BP: Biological process
CC: Cell component
MF: Molecular function
KEGG: Kyoto Encyclopedia of genes and Genomes
MCODE: Minimal Common Oncology Data Elements
DO: Disease ontology
GSEA: Gene Set Enrichment Analysis
ROC: Receiver operator characteristic curve
AUC: Area under the curve
lncRNA: Long non-coding RNA
CAMs: Cell adhesion molecules
SLC6A14: Sodium- and chloride-dependent neutral and basic amino acid transporter B
TIMP1: Metalloproteinase inhibitor
APOBEC3B: DNA dC- > dU-editing enzyme
IRAK3: Interleukin-1 receptor-associated kinase 3
HMGCS2: Hydroxymethylglutaryl-CoA synthase
NK: Natural killing
NORAD: Non-coding RNA activated by DNA damage
OIP5-AS1: OIP5 antisense RNA1
XIST: X inactive specific transcript
MALAT1: Metastasis associated lung adenocarcinoma transcript 1
NEAT1: Nuclear paraspeckle assembly transcript 1
PAR: Protease activated receptor
CRC: Colorectal cancer
MMPs: Matrix metalloproteinase
