# Supplementary material for: Screening of ulcerative colitis biomarkers and potential pathways based on weighted gene co-expression network, machine learning and ceRNA hypothesis
Source: Hereditas. 2022 Nov 23;159:42. doi: 10.1186/s41065-022-00259-4 (PMC9685902; doi:10.1186/s41065-022-00259-4)
Supplement: Supplementary file 1 — Additional file 1. The Supplementary Material for this article can be found online at Mendeley Data, V1, DOI:https://doi.org/10.17632/58hf8kz9tm.1 [file 41065_2022_259_MOESM1_ESM.docx]

**S1 Table. Results of co-expression of miRNAs with five biomarkers.** The table shows the results of co-expressed miRNAs for 5 potential biomarkers, with a total of 54 co-expressed miRNAs.

| **mRNAs** | **miRNA** |
| --- | --- |
| HMGCS2-SLC6A14 | hsa-miR-103a-3p |
| HMGCS2-SLC6A14 | hsa-miR-107 |
| HMGCS2-SLC6A14 | hsa-miR-135a-5p |
| HMGCS2-SLC6A14 | hsa-miR-135b-5p |
| HMGCS2-SLC6A14 | hsa-miR-139-5p |
| HMGCS2-SLC6A14 | hsa-miR-142-5p |
| HMGCS2-SLC6A14 | hsa-miR-21-5p |
| HMGCS2-SLC6A14 | hsa-miR-25-3p |
| HMGCS2-SLC6A14 | hsa-miR-27a-3p |
| HMGCS2-SLC6A14 | hsa-miR-27b-3p |
| HMGCS2-SLC6A14 | hsa-miR-29a-3p |
| HMGCS2-SLC6A14 | hsa-miR-29b-3p |
| HMGCS2-SLC6A14 | hsa-miR-29c-3p |
| HMGCS2-SLC6A14 | hsa-miR-32-5p |
| HMGCS2-SLC6A14 | hsa-miR-363-3p |
| HMGCS2-SLC6A14 | hsa-miR-367-3p |
| HMGCS2-SLC6A14 | hsa-miR-513b-5p |
| HMGCS2-SLC6A14 | hsa-miR-5590-3p |
| HMGCS2-SLC6A14 | hsa-miR-590-5p |
| HMGCS2-SLC6A14 | hsa-miR-624-3p |
| HMGCS2-SLC6A14 | hsa-miR-656-3p |
| HMGCS2-SLC6A14 | hsa-miR-92a-3p |
| HMGCS2-SLC6A14 | hsa-miR-92b-3p |
| HMGCS2-SLC6A14 | hsa-miR-9-5p |
| SLC6A14-TIMP1 | hsa-miR-196a-5p |
| SLC6A14-TIMP1 | hsa-miR-22-3p |
| SLC6A14-TIMP1 | hsa-miR-33b-5p |
| SLC6A14-TIMP1 | hsa-miR-503-5p |
| SLC6A14-TIMP1 | hsa-miR-548o-3p |
| SLC6A14-TIMP1 | hsa-miR-663a |
| SLC6A14-IRAK3 | hsa-miR-1224-5p |
| SLC6A14-IRAK3 | hsa-miR-2467-3p |
| SLC6A14-IRAK3 | hsa-miR-300 |
| SLC6A14-IRAK3 | hsa-miR-323a-3p |
| SLC6A14-IRAK3 | hsa-miR-342-3p |
| SLC6A14-IRAK3 | hsa-miR-377-3p |
| SLC6A14-IRAK3 | hsa-miR-381-3p |
| SLC6A14-IRAK3 | hsa-miR-384 |
| SLC6A14-IRAK3 | hsa-miR-409-3p |
| SLC6A14-IRAK3 | hsa-miR-410-3p |
| SLC6A14-IRAK3 | hsa-miR-485-5p |
| SLC6A14-IRAK3 | hsa-miR-556-5p |
| SLC6A14-IRAK3 | hsa-miR-650 |
| HMGCS2-IRAK3 | hsa-miR-1270 |
| HMGCS2-IRAK3 | hsa-miR-185-5p |
| HMGCS2-IRAK3 | hsa-miR-1197 |
| HMGCS2-IRAK3 | hsa-miR-620 |
| TIMP1-IRAK3 | hsa-miR-1321 |
| TIMP1-IRAK3 | hsa-miR-4756-5p |
| SLC6A14-APOBEC3B | hsa-miR-205-5p |
| HMGCS2-SLC6A14-IRAK3 | hsa-miR-520d-5p |
| HMGCS2-SLC6A14-IRAK3 | hsa-miR-7-5p |
| HMGCS2-SLC6A14-IRAK3 | hsa-miR-524-5p |
| HMGCS2-SLC6A14-IRAK3 | hsa-miR-513a-5p |

**S2 Table. miRNAs and lncRNAs associated with five potential biomarkers.** The table shows the miRNAs and lncRNAs associated with five potential biomarkers predicted by the database. The lncRANs were predicted based on the co-expressed 54 miRNAs.

| **mRNA** | **miRNA** | **lncRNA** |
| --- | --- | --- |
| HMGCS2 | hsa-miR-135a-5p | NEAT1 |
| HMGCS2 | hsa-miR-135b-5p | NEAT1 |
| HMGCS2 | hsa-miR-139-5p | NEAT1 |
| HMGCS2 | hsa-miR-142-5p | NEAT1 |
| HMGCS2 | hsa-miR-27a-3p | NEAT1 |
| HMGCS2 | hsa-miR-27b-3p | NEAT1 |
| HMGCS2 | hsa-miR-29a-3p | NEAT1 |
| HMGCS2 | hsa-miR-29b-3p | NEAT1 |
| HMGCS2 | hsa-miR-29c-3p | NEAT1 |
| HMGCS2 | hsa-miR-513b-5p | NEAT1 |
| HMGCS2 | hsa-miR-5590-3p | NEAT1 |
| HMGCS2 | hsa-miR-624-3p | NEAT1 |
| HMGCS2 | hsa-miR-656-3p | NEAT1 |
| HMGCS2 | hsa-miR-103a-3p | NEAT1 |
| HMGCS2 | hsa-miR-107 | NEAT1 |
| HMGCS2 | hsa-miR-9-5p | NEAT1 |
| HMGCS2 | hsa-miR-1270 | NEAT1 |
| HMGCS2 | hsa-miR-185-5p | NEAT1 |
| HMGCS2 | hsa-miR-1197 | NEAT1 |
| HMGCS2 | hsa-miR-620 | NEAT1 |
| HMGCS2 | hsa-miR-520d-5p | NEAT1 |
| HMGCS2 | hsa-miR-524-5p | NEAT1 |
| HMGCS2 | hsa-miR-513a-5p | NEAT1 |
| HMGCS2 | hsa-miR-103a-3p | XIST |
| HMGCS2 | hsa-miR-107 | XIST |
| HMGCS2 | hsa-miR-135a-5p | XIST |
| HMGCS2 | hsa-miR-135b-5p | XIST |
| HMGCS2 | hsa-miR-139-5p | XIST |
| HMGCS2 | hsa-miR-142-5p | XIST |
| HMGCS2 | hsa-miR-21-5p | XIST |
| HMGCS2 | hsa-miR-25-3p | XIST |
| HMGCS2 | hsa-miR-27a-3p | XIST |
| HMGCS2 | hsa-miR-27b-3p | XIST |
| HMGCS2 | hsa-miR-29a-3p | XIST |
| HMGCS2 | hsa-miR-29b-3p | XIST |
| HMGCS2 | hsa-miR-29c-3p | XIST |
| HMGCS2 | hsa-miR-32-5p | XIST |
| HMGCS2 | hsa-miR-363-3p | XIST |
| HMGCS2 | hsa-miR-367-3p | XIST |
| HMGCS2 | hsa-miR-513b-5p | XIST |
| HMGCS2 | hsa-miR-5590-3p | XIST |
| HMGCS2 | hsa-miR-590-5p | XIST |
| HMGCS2 | hsa-miR-624-3p | XIST |
| HMGCS2 | hsa-miR-656-3p | XIST |
| HMGCS2 | hsa-miR-92a-3p | XIST |
| HMGCS2 | hsa-miR-92b-3p | XIST |
| HMGCS2 | hsa-miR-9-5p | XIST |
| HMGCS2 | hsa-miR-1270 | XIST |
| HMGCS2 | hsa-miR-185-5p | XIST |
| HMGCS2 | hsa-miR-1197 | XIST |
| HMGCS2 | hsa-miR-620 | XIST |
| HMGCS2 | hsa-miR-520d-5p | XIST |
| HMGCS2 | hsa-miR-524-5p | XIST |
| HMGCS2 | hsa-miR-135a-5p | OIP5-AS1 |
| HMGCS2 | hsa-miR-135b-5p | OIP5-AS1 |
| HMGCS2 | hsa-miR-142-5p | OIP5-AS1 |
| HMGCS2 | hsa-miR-25-3p | OIP5-AS1 |
| HMGCS2 | hsa-miR-29a-3p | OIP5-AS1 |
| HMGCS2 | hsa-miR-29b-3p | OIP5-AS1 |
| HMGCS2 | hsa-miR-29c-3p | OIP5-AS1 |
| HMGCS2 | hsa-miR-32-5p | OIP5-AS1 |
| HMGCS2 | hsa-miR-363-3p | OIP5-AS1 |
| HMGCS2 | hsa-miR-367-3p | OIP5-AS1 |
| HMGCS2 | hsa-miR-513b-5p | OIP5-AS1 |
| HMGCS2 | hsa-miR-5590-3p | OIP5-AS1 |
| HMGCS2 | hsa-miR-92a-3p | OIP5-AS1 |
| HMGCS2 | hsa-miR-92b-3p | OIP5-AS1 |
| HMGCS2 | hsa-miR-1270 | OIP5-AS1 |
| HMGCS2 | hsa-miR-1197 | OIP5-AS1 |
| HMGCS2 | hsa-miR-620 | OIP5-AS1 |
| HMGCS2 | hsa-miR-513a-5p | OIP5-AS1 |
| HMGCS2 | hsa-miR-25-3p | NORAD |
| HMGCS2 | hsa-miR-32-5p | NORAD |
| HMGCS2 | hsa-miR-363-3p | NORAD |
| HMGCS2 | hsa-miR-367-3p | NORAD |
| HMGCS2 | hsa-miR-513b-5p | NORAD |
| HMGCS2 | hsa-miR-656-3p | NORAD |
| HMGCS2 | hsa-miR-92a-3p | NORAD |
| HMGCS2 | hsa-miR-92b-3p | NORAD |
| HMGCS2 | hsa-miR-1270 | NORAD |
| HMGCS2 | hsa-miR-620 | NORAD |
| HMGCS2 | hsa-miR-520d-5p | NORAD |
| HMGCS2 | hsa-miR-524-5p | NORAD |
| HMGCS2 | hsa-miR-135a-5p | MALAT1 |
| HMGCS2 | hsa-miR-135b-5p | MALAT1 |
| HMGCS2 | hsa-miR-25-3p | MALAT1 |
| HMGCS2 | hsa-miR-32-5p | MALAT1 |
| HMGCS2 | hsa-miR-363-3p | MALAT1 |
| HMGCS2 | hsa-miR-367-3p | MALAT1 |
| HMGCS2 | hsa-miR-92a-3p | MALAT1 |
| HMGCS2 | hsa-miR-92b-3p | MALAT1 |
| HMGCS2 | hsa-miR-1270 | MALAT1 |
| HMGCS2 | hsa-miR-185-5p | MALAT1 |
| HMGCS2 | hsa-miR-620 | MALAT1 |
| SLC6A14 | hsa-miR-135a-5p | NEAT1 |
| SLC6A14 | hsa-miR-135b-5p | NEAT1 |
| SLC6A14 | hsa-miR-139-5p | NEAT1 |
| SLC6A14 | hsa-miR-142-5p | NEAT1 |
| SLC6A14 | hsa-miR-27a-3p | NEAT1 |
| SLC6A14 | hsa-miR-27b-3p | NEAT1 |
| SLC6A14 | hsa-miR-29a-3p | NEAT1 |
| SLC6A14 | hsa-miR-29b-3p | NEAT1 |
| SLC6A14 | hsa-miR-29c-3p | NEAT1 |
| SLC6A14 | hsa-miR-513b-5p | NEAT1 |
| SLC6A14 | hsa-miR-5590-3p | NEAT1 |
| SLC6A14 | hsa-miR-624-3p | NEAT1 |
| SLC6A14 | hsa-miR-656-3p | NEAT1 |
| SLC6A14 | hsa-miR-103a-3p | NEAT1 |
| SLC6A14 | hsa-miR-107 | NEAT1 |
| SLC6A14 | hsa-miR-9-5p | NEAT1 |
| SLC6A14 | hsa-miR-196a-5p | NEAT1 |
| SLC6A14 | hsa-miR-22-3p | NEAT1 |
| SLC6A14 | hsa-miR-33b-5p | NEAT1 |
| SLC6A14 | hsa-miR-503-5p | NEAT1 |
| SLC6A14 | hsa-miR-548o-3p | NEAT1 |
| SLC6A14 | hsa-miR-663a | NEAT1 |
| SLC6A14 | hsa-miR-1224-5p | NEAT1 |
| SLC6A14 | hsa-miR-2467-3p | NEAT1 |
| SLC6A14 | hsa-miR-300 | NEAT1 |
| SLC6A14 | hsa-miR-323a-3p | NEAT1 |
| SLC6A14 | hsa-miR-342-3p | NEAT1 |
| SLC6A14 | hsa-miR-377-3p | NEAT1 |
| SLC6A14 | hsa-miR-381-3p | NEAT1 |
| SLC6A14 | hsa-miR-384 | NEAT1 |
| SLC6A14 | hsa-miR-409-3p | NEAT1 |
| SLC6A14 | hsa-miR-410-3p | NEAT1 |
| SLC6A14 | hsa-miR-485-5p | NEAT1 |
| SLC6A14 | hsa-miR-556-5p | NEAT1 |
| SLC6A14 | hsa-miR-650 | NEAT1 |
| SLC6A14 | hsa-miR-205-5p | NEAT1 |
| SLC6A14 | hsa-miR-513a-5p | NEAT1 |
| SLC6A14 | hsa-miR-520d-5p | NEAT1 |
| SLC6A14 | hsa-miR-524-5p | NEAT1 |
| SLC6A14 | hsa-miR-103a-3p | XIST |
| SLC6A14 | hsa-miR-107 | XIST |
| SLC6A14 | hsa-miR-135a-5p | XIST |
| SLC6A14 | hsa-miR-135b-5p | XIST |
| SLC6A14 | hsa-miR-139-5p | XIST |
| SLC6A14 | hsa-miR-142-5p | XIST |
| SLC6A14 | hsa-miR-21-5p | XIST |
| SLC6A14 | hsa-miR-25-3p | XIST |
| SLC6A14 | hsa-miR-27a-3p | XIST |
| SLC6A14 | hsa-miR-27b-3p | XIST |
| SLC6A14 | hsa-miR-29a-3p | XIST |
| SLC6A14 | hsa-miR-29b-3p | XIST |
| SLC6A14 | hsa-miR-29c-3p | XIST |
| SLC6A14 | hsa-miR-32-5p | XIST |
| SLC6A14 | hsa-miR-363-3p | XIST |
| SLC6A14 | hsa-miR-367-3p | XIST |
| SLC6A14 | hsa-miR-513b-5p | XIST |
| SLC6A14 | hsa-miR-5590-3p | XIST |
| SLC6A14 | hsa-miR-590-5p | XIST |
| SLC6A14 | hsa-miR-624-3p | XIST |
| SLC6A14 | hsa-miR-656-3p | XIST |
| SLC6A14 | hsa-miR-92a-3p | XIST |
| SLC6A14 | hsa-miR-92b-3p | XIST |
| SLC6A14 | hsa-miR-9-5p | XIST |
| SLC6A14 | hsa-miR-196a-5p | XIST |
| SLC6A14 | hsa-miR-663a | XIST |
| SLC6A14 | hsa-miR-1224-5p | XIST |
| SLC6A14 | hsa-miR-2467-3p | XIST |
| SLC6A14 | hsa-miR-300 | XIST |
| SLC6A14 | hsa-miR-323a-3p | XIST |
| SLC6A14 | hsa-miR-342-3p | XIST |
| SLC6A14 | hsa-miR-377-3p | XIST |
| SLC6A14 | hsa-miR-381-3p | XIST |
| SLC6A14 | hsa-miR-410-3p | XIST |
| SLC6A14 | hsa-miR-485-5p | XIST |
| SLC6A14 | hsa-miR-7-5p | XIST |
| SLC6A14 | hsa-miR-524-5p | XIST |
| SLC6A14 | hsa-miR-520d-5p | XIST |
| SLC6A14 | hsa-miR-135a-5p | OIP5-AS1 |
| SLC6A14 | hsa-miR-135b-5p | OIP5-AS1 |
| SLC6A14 | hsa-miR-142-5p | OIP5-AS1 |
| SLC6A14 | hsa-miR-25-3p | OIP5-AS1 |
| SLC6A14 | hsa-miR-29a-3p | OIP5-AS1 |
| SLC6A14 | hsa-miR-29b-3p | OIP5-AS1 |
| SLC6A14 | hsa-miR-29c-3p | OIP5-AS1 |
| SLC6A14 | hsa-miR-32-5p | OIP5-AS1 |
| SLC6A14 | hsa-miR-363-3p | OIP5-AS1 |
| SLC6A14 | hsa-miR-367-3p | OIP5-AS1 |
| SLC6A14 | hsa-miR-513b-5p | OIP5-AS1 |
| SLC6A14 | hsa-miR-5590-3p | OIP5-AS1 |
| SLC6A14 | hsa-miR-92a-3p | OIP5-AS1 |
| SLC6A14 | hsa-miR-92b-3p | OIP5-AS1 |
| SLC6A14 | hsa-miR-22-3p | OIP5-AS1 |
| SLC6A14 | hsa-miR-548o-3p | OIP5-AS1 |
| SLC6A14 | hsa-miR-300 | OIP5-AS1 |
| SLC6A14 | hsa-miR-342-3p | OIP5-AS1 |
| SLC6A14 | hsa-miR-381-3p | OIP5-AS1 |
| SLC6A14 | hsa-miR-410-3p | OIP5-AS1 |
| SLC6A14 | hsa-miR-513a-5p | OIP5-AS1 |
| SLC6A14 | hsa-miR-7-5p | OIP5-AS1 |
| SLC6A14 | hsa-miR-25-3p | NORAD |
| SLC6A14 | hsa-miR-32-5p | NORAD |
| SLC6A14 | hsa-miR-363-3p | NORAD |
| SLC6A14 | hsa-miR-367-3p | NORAD |
| SLC6A14 | hsa-miR-513b-5p | NORAD |
| SLC6A14 | hsa-miR-656-3p | NORAD |
| SLC6A14 | hsa-miR-92a-3p | NORAD |
| SLC6A14 | hsa-miR-92b-3p | NORAD |
| SLC6A14 | hsa-miR-22-3p | NORAD |
| SLC6A14 | hsa-miR-663a | NORAD |
| SLC6A14 | hsa-miR-205-5p | NORAD |
| SLC6A14 | hsa-miR-2467-3p | NORAD |
| SLC6A14 | hsa-miR-323a-3p | NORAD |
| SLC6A14 | hsa-miR-377-3p | NORAD |
| SLC6A14 | hsa-miR-384 | NORAD |
| SLC6A14 | hsa-miR-410-3p | NORAD |
| SLC6A14 | hsa-miR-520d-5p | NORAD |
| SLC6A14 | hsa-miR-524-5p | NORAD |
| SLC6A14 | hsa-miR-32-5p | MALAT1 |
| SLC6A14 | hsa-miR-363-3p | MALAT1 |
| SLC6A14 | hsa-miR-367-3p | MALAT1 |
| SLC6A14 | hsa-miR-92a-3p | MALAT1 |
| SLC6A14 | hsa-miR-92b-3p | MALAT1 |
| SLC6A14 | hsa-miR-22-3p | MALAT1 |
| SLC6A14 | hsa-miR-503-5p | MALAT1 |
| SLC6A14 | hsa-miR-548o-3p | MALAT1 |
| SLC6A14 | hsa-miR-384 | MALAT1 |
| SLC6A14 | hsa-miR-485-5p | MALAT1 |
| SLC6A14 | hsa-miR-205-5p | MALAT1 |
| IRAK3 | hsa-miR-1224-5p | NEAT1 |
| IRAK3 | hsa-miR-2467-3p | NEAT1 |
| IRAK3 | hsa-miR-300 | NEAT1 |
| IRAK3 | hsa-miR-323a-3p | NEAT1 |
| IRAK3 | hsa-miR-342-3p | NEAT1 |
| IRAK3 | hsa-miR-377-3p | NEAT1 |
| IRAK3 | hsa-miR-381-3p | NEAT1 |
| IRAK3 | hsa-miR-384 | NEAT1 |
| IRAK3 | hsa-miR-409-3p | NEAT1 |
| IRAK3 | hsa-miR-410-3p | NEAT1 |
| IRAK3 | hsa-miR-485-5p | NEAT1 |
| IRAK3 | hsa-miR-556-5p | NEAT1 |
| IRAK3 | hsa-miR-650 | NEAT1 |
| IRAK3 | hsa-miR-1270 | NEAT1 |
| IRAK3 | hsa-miR-185-5p | NEAT1 |
| IRAK3 | hsa-miR-1197 | NEAT1 |
| IRAK3 | hsa-miR-620 | NEAT1 |
| IRAK3 | hsa-miR-1321 | NEAT1 |
| IRAK3 | hsa-miR-4756-5p | NEAT1 |
| IRAK3 | hsa-miR-520d-5p | NEAT1 |
| IRAK3 | hsa-miR-524-5p | NEAT1 |
| IRAK3 | hsa-miR-1270 | XIST |
| IRAK3 | hsa-miR-185-5p | XIST |
| IRAK3 | hsa-miR-1197 | XIST |
| IRAK3 | hsa-miR-620 | XIST |
| IRAK3 | hsa-miR-1224-5p | XIST |
| IRAK3 | hsa-miR-2467-3p | XIST |
| IRAK3 | hsa-miR-300 | XIST |
| IRAK3 | hsa-miR-323a-3p | XIST |
| IRAK3 | hsa-miR-342-3p | XIST |
| IRAK3 | hsa-miR-377-3p | XIST |
| IRAK3 | hsa-miR-381-3p | XIST |
| IRAK3 | hsa-miR-410-3p | XIST |
| IRAK3 | hsa-miR-485-5p | XIST |
| IRAK3 | hsa-miR-1321 | XIST |
| IRAK3 | hsa-miR-4756-5p | XIST |
| IRAK3 | hsa-miR-7-5p | XIST |
| IRAK3 | hsa-miR-520d-5p | XIST |
| IRAK3 | hsa-miR-524-5p | XIST |
| IRAK3 | hsa-miR-300 | OIP5-AS1 |
| IRAK3 | hsa-miR-342-3p | OIP5-AS1 |
| IRAK3 | hsa-miR-381-3p | OIP5-AS1 |
| IRAK3 | hsa-miR-410-3p | OIP5-AS1 |
| IRAK3 | hsa-miR-1270 | OIP5-AS1 |
| IRAK3 | hsa-miR-1197 | OIP5-AS1 |
| IRAK3 | hsa-miR-620 | OIP5-AS1 |
| IRAK3 | hsa-miR-1321 | OIP5-AS1 |
| IRAK3 | hsa-miR-4756-5p | OIP5-AS1 |
| IRAK3 | hsa-miR-7-5p | OIP5-AS1 |
| IRAK3 | hsa-miR-2467-3p | NORAD |
| IRAK3 | hsa-miR-323a-3p | NORAD |
| IRAK3 | hsa-miR-377-3p | NORAD |
| IRAK3 | hsa-miR-384 | NORAD |
| IRAK3 | hsa-miR-410-3p | NORAD |
| IRAK3 | hsa-miR-1270 | NORAD |
| IRAK3 | hsa-miR-620 | NORAD |
| IRAK3 | hsa-miR-520d-5p | NORAD |
| IRAK3 | hsa-miR-524-5p | NORAD |
| IRAK3 | hsa-miR-384 | MALAT1 |
| IRAK3 | hsa-miR-485-5p | MALAT1 |
| IRAK3 | hsa-miR-1270 | MALAT1 |
| IRAK3 | hsa-miR-185-5p | MALAT1 |
| IRAK3 | hsa-miR-620 | MALAT1 |
| IRAK3 | hsa-miR-1321 | MALAT1 |
| IRAK3 | hsa-miR-4756-5p | MALAT1 |
| TIMP1 | hsa-miR-196a-5p | NEAT1 |
| TIMP1 | hsa-miR-22-3p | NEAT1 |
| TIMP1 | hsa-miR-33b-5p | NEAT1 |
| TIMP1 | hsa-miR-503-5p | NEAT1 |
| TIMP1 | hsa-miR-548o-3p | NEAT1 |
| TIMP1 | hsa-miR-663a | NEAT1 |
| TIMP1 | hsa-miR-1321 | NEAT1 |
| TIMP1 | hsa-miR-4756-5p | NEAT1 |
| TIMP1 | hsa-miR-513a-5p | NEAT1 |
| TIMP1 | hsa-miR-196a-5p | XIST |
| TIMP1 | hsa-miR-663a | XIST |
| TIMP1 | hsa-miR-7-5p | XIST |
| TIMP1 | hsa-miR-1321 | XIST |
| TIMP1 | hsa-miR-4756-5p | XIST |
| TIMP1 | hsa-miR-1321 | OIP5-AS1 |
| TIMP1 | hsa-miR-4756-5p | OIP5-AS1 |
| TIMP1 | hsa-miR-22-3p | OIP5-AS1 |
| TIMP1 | hsa-miR-548o-3p | OIP5-AS1 |
| TIMP1 | hsa-miR-7-5p | OIP5-AS1 |
| TIMP1 | hsa-miR-513a-5p | OIP5-AS1 |
| TIMP1 | hsa-miR-22-3p | NORAD |
| TIMP1 | hsa-miR-663a | NORAD |
| TIMP1 | hsa-miR-22-3p | MALAT1 |
| TIMP1 | hsa-miR-503-5p | MALAT1 |
| TIMP1 | hsa-miR-548o-3p | MALAT1 |
| TIMP1 | hsa-miR-1321 | MALAT1 |
| TIMP1 | hsa-miR-4756-5p | MALAT1 |
| APOBEC3B | hsa-miR-205-5p | NEAT1 |
| APOBEC3B | hsa-miR-205-5p | MALAT1 |
| APOBEC3B | hsa-miR-205-5p | NORAD |
